# AIP4/Itch Regulates Notch Receptor Degradation in the Absence of Ligand

**DOI:** 10.1371/journal.pone.0002735

**Published:** 2008-07-16

**Authors:** Patricia Chastagner, Alain Israël, Christel Brou

**Affiliations:** Unité de Signalisation Moléculaire et Activation Cellulaire, URA 2582, CNRS, Institut Pasteur, Paris, France; Universität Heidelberg, Germany

## Abstract

**Background:**

The regulation of Notch signaling heavily relies on ubiquitination events. Drosophila Su(dx), a member of the HECT family of ubiquitin-ligases, has been described as a negative regulator of Notch signaling, acting on the post-endocytic sorting of Notch. The mammalian ortholog of Su(dx), Itch/AIP4, has been shown to have multiple substrates, including Notch, but the precise events regulated by Itch/AIP4 in the Notch pathway have not been identified yet.

**Methodology/Principal Findings:**

Using Itch-/- fibroblasts expressing the Notch1 receptor, we show that Itch is not necessary for Notch activation, but rather for controlling the degradation of Notch in the absence of ligand. Itch is indeed required after the early steps of Notch endocytosis to target it to the lysosomes where it is degraded. Furthermore Itch/AIP4 catalyzes Notch polyubiquitination through unusual K29-linked chains. We also demonstrate that although Notch is associated with Itch/AIP4 in cells, their interaction is not detectable *in vitro* and thus requires either a post-translational modification, or a bridging factor that remains to be identified.

**Conclusions/Significance:**

Taken together our results identify a specific step of Notch regulation in the absence of any activation and underline differences between mammalian and Drosophila Notch pathways.

## Introduction

Notch signaling is an evolutionarily conserved process involved in cell fate decisions, cell proliferation or cell death, during development and in the adulthood, and its deregulation leads to several pathologies in mammals. The canonical Notch activation pathway relies on the regulated proteolysis of this membrane receptor after binding to one of its ligands. The resulting free intracellular domain of Notch (ICv) translocates to the nucleus to form a transcriptional activation complex with other cofactors [1]. Besides the various combinations of ligands and receptors probably leading to various transcriptional responses, the quantitative aspects of the signaling pathway have to be controlled. These regulations may occur at the level of the production and/or stability of ICv, affinity of the receptor for its ligand, and quantity of ligand and receptor present at the cell surface.

Suppressor of deltex (Su(dx)) was first described in Drosophila as a negative regulator of Notch signaling, acting in an antagonist manner to Deltex (dx), another component of the pathway [2]–[4]. The phenotypes resulting from the overexpression of the Su(dx) protein in the developing wing were those expected from a downregulation of the Notch pathway. Mammalian orthologs of Su(dx) are called Itch in the mouse and AIP4 in humans. Mammalian Itch was identified in natural mutant mice (itchy mice, [5]) that develop a progressive autoimmune-like disease, partly because Itch targets such as junB are relevant to autoimmunity. However it was recently shown that increased Notch signaling in transgenic mice mimics the symptoms of the disease [6]. Various substrates have been described for Itch in mammals: CXCR4 [7], p73, p63 [8], [9] smad 7 [10], Jun [11], Deltex (DTX, [12]) and Endophilin [13]. In general Itch targets its substrates to degradation, with some exceptions: AIP4 regulates the cell surface expression of select TRP channels by enhancing their ubiquitination and endocytosis but without facilitating their degradation [14]. Itch activity on junB is enhanced by Ser/Thr phosphorylation by MEKK1-JNK1 kinases [11], and reduced by Tyr phosphorylation in a fyn-dependent manner [15]. Thus it is difficult to attribute general characteristics to Itch, except that it is located in the endosomal system [7], [13], [16] and that it is autoubiquitinated [12], [17]. Furthermore the type of chains formed on its substrates is not often identified (except for K29-linked polyubiquitin chains on DTX and itself, [12]). Even less is known in Drosophila about the mechanisms controlled by Su(dx), Notch being its unique described target in this organism. Sakata et al. [18] have shown that ubiquitination of Drosophila Notch depends on Nedd4 (an E3 ubiquitin ligase belonging to the same family as Su(dx)) and on the presence of a PPSY motif in the intracellular region of Notch. Nedd4 is involved in the constitutive endocytosis of Notch and regulates its stability. Wilkin et al. [19] have demonstrated that Su(dx) and/or Nedd4 regulate sorting of Notch full-length within the early endosome. However these authors did not determine whether ubiquitination of Notch targets it for degradation, recycling or some other fate.

In mammals, Qiu et al [20] have shown that Itch is able to direct the ubiquitination of Notch ΔE (a constitutively active but membrane-anchored form of Notch). Based on interaction experiments, they concluded that the Notch IC domain is a direct substrate for Itch. However the step regulated by Itch was not clearly defined.

As these conclusions on the role of Itch on an activated form of mammalian Notch contradict the observations made in Drosophila, we decided to identify the form of Notch that was targeted by Itch, and to characterize the consequences of this ubiquitination.

We demonstrate here that Itch controls the degradation of the non-activated receptor, inducing after early endocytosis the formation of K29-linked polyubiquitin chains and targeting Notch to lysosomal degradation. We also show that Itch does not interact directly with Notch in mammals and might require a bridging factor.

## Results

### Notch activation does not depend on Itch

**Figure 1:**
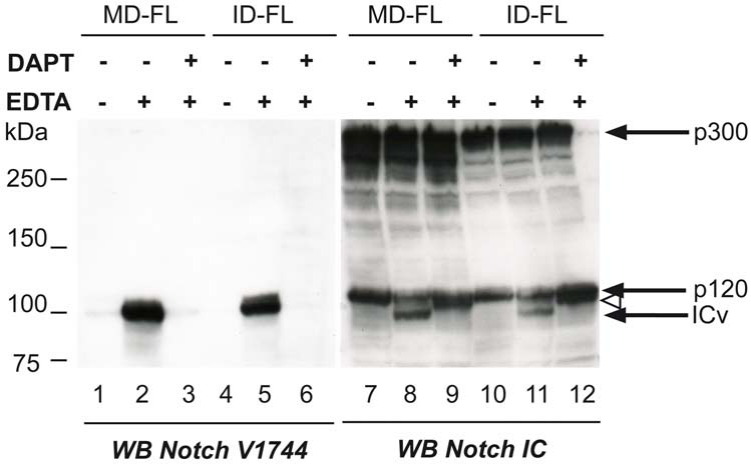
Notch activation by EDTA in WT (MD-FL) and Itch-/- (ID-FL) cells. Cells were activated by a 15 minutes EDTA treatment, in the presence of DAPT when indicated. Extracts were then directly prepared and tested by immunoblotting with antibodies recognizing the activated form of Notch (V1744 antibody, lanes 1 to 6), or the whole intracellular domain (Notch IC antibody, lanes 7 to 12). The open arrowhead indicates the S2 cleavage product appearing after Notch activation. p300 and p120 represent respectively the Notch precursor and its furin-generated transmembrane product.

It has been shown in Drosophila that Notch signaling is limited by the activity of Su(dx) and DNedd4. In addition the inhibition of Nedd4 activity leads to ligand-independent activation of Notch [18]. To test whether the absence of Itch had any consequence on Notch activation in a mammalian system, we transduced MEFs with retroviral vectors to allow expression of human Notch 1 at the cell surface. In this vector an HA tag sequence has been inserted between EGF repeats 22 and 23 of Notch [21]. These cells, either WT (called MD-FL) or Itch-/- (ID-FL), have also been stably transfected by a VSV-tagged version of hDTX1 [12]. Notch activation was obtained by separation of its heterodimeric form following calcium depletion by EDTA [21]. This well documented method allows the rapid and efficient activation of Notch throughout a cell population. Notch activation was monitored by the presence of the Notch intracellular fragment (ICv) in the extracts, detected with the specific V1744 N-terminal antibody (Figure 1). No activated form was detected in the extracts derived from both cell lines, showing that Notch was not constitutively activated in Itch-/- cells (lanes 1 and 4). EDTA treatment led to the production of comparable amounts of ICv in both cell lines (lanes 2 and 5). ICv was also clearly detectable by direct blotting with an anti-Notch IC antibody (compare lanes 8, 11 to 7, 10). EDTA activation of Notch was dependent both on γ-secretase activity, since DAPT (a γ-secretase inhibitor) treatment abolishes ICv production (lanes 3, 6), and on the membrane metalloprotease TACE, since the S2 cleavage product accumulated in the presence of DAPT (lanes 9, 12, open arrowhead). Thus in the absence of Itch, Notch receptor was fully functional and no ectopic activation was observed. Therefore the increased Notch signaling in Itch -/- mice [6] might be rather due to a defect in its negative regulation.

### Notch receptor in the absence of ligand is targeted to lysosomal degradation in an Itch-dependent manner

**Figure 2:**
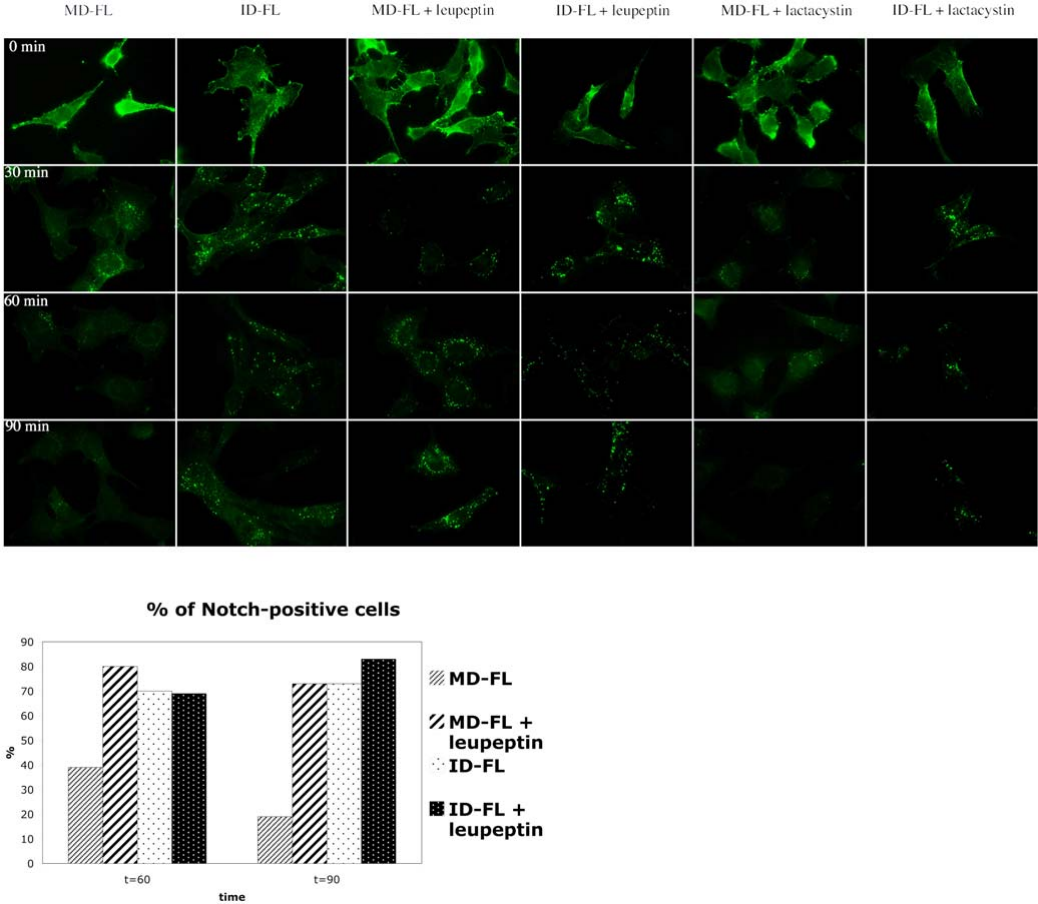
Notch endocytosis and degradation. Pulse-chase antibody uptake assay on MD-FL or ID-FL cells. Alexa 488-coupled anti-HA antibody (green) was taken into the cells by endocytosis during various periods of time at 37°C. When indicated, cells were incubated 1 h before adding antibody with leupeptin or lactacystin, and maintained during the entire experiment in the presence of these inhibitors. The data are representative of 4 independent experiments. Bottom, quantification of Notch degradation: the percentage of cells showing HA staining after 60 or 90 minutes of incubation was calculated as an average from at least 40 cells providing from 4 independent experiments.

**Figure 3:**
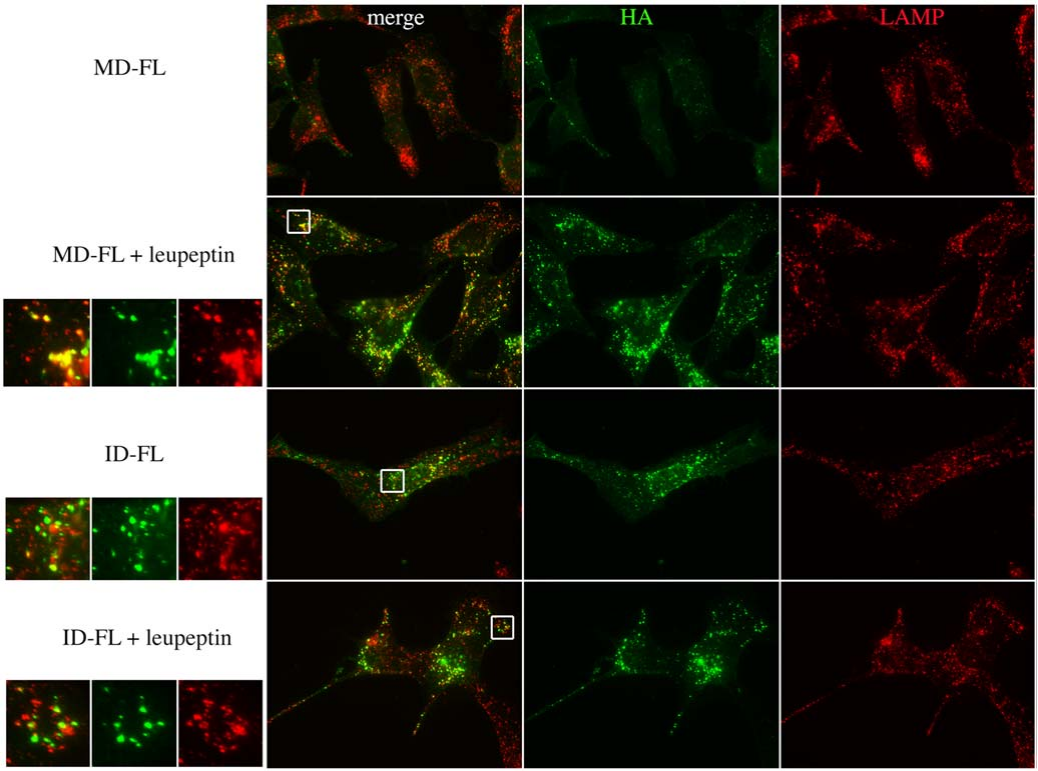
Notch degradation occurs in the lysosomes. The anti-HA antibody uptake assay was performed as in figure 2. After 90 minutes of chase, cells were fixed, permeabilized and incubated with an anti-LAMP-1 antibody, followed by a mouse-adsorbed rabbit anti-rat and then Al555-coupled anti-rabbit antibodies. Insets represent enlargements (fourfold) of the boxed regions. The photographs are representative of a large number of observed fields. The relative amount of Notch-containing vesicles also positive for LAMP-1 was calculated from 10 cells in each condition. In average less than 25% of Notch vesicles were LAMP-1 negative in MD-FL+Leupeptin, whereas less than 25% of Notch vesicles were positive for LAMP-1 in ID-FL cells (+/− Leupeptin).

**Figure 4:**
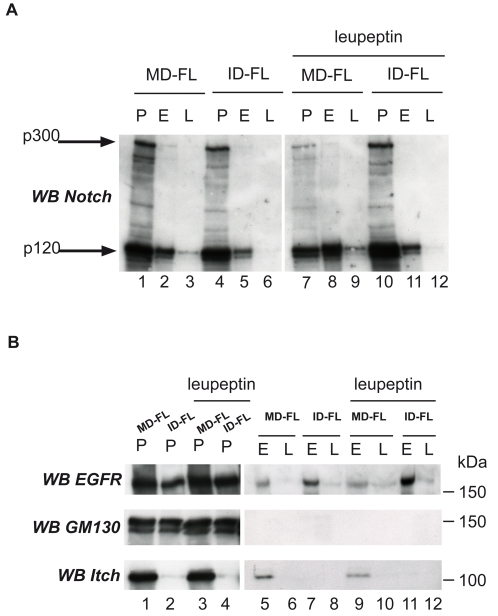
Subcellular fractionation of Notch-expressing cells. MD-FL or ID-FL cells (non-treated or after leupeptin treatment as indicated) were mechanically lysed and a post-nuclear supernatant (P) was prepared. Early (E) or late (L) endosomes were prepared by floatation in a sucrose gradient, and blotted for the presence of Notch in A. p300 is the full-length Notch molecule retained in the Golgi apparatus before its maturation by furin protease, p120 is the mature membrane-associated C-terminal part of the molecule. In B, the fractions were analyzed with rabbit anti-EGFR, anti-GM130 (as a Golgi marker) and monoclonal anti-Itch, as indicated on the left.

**Figure 5:**
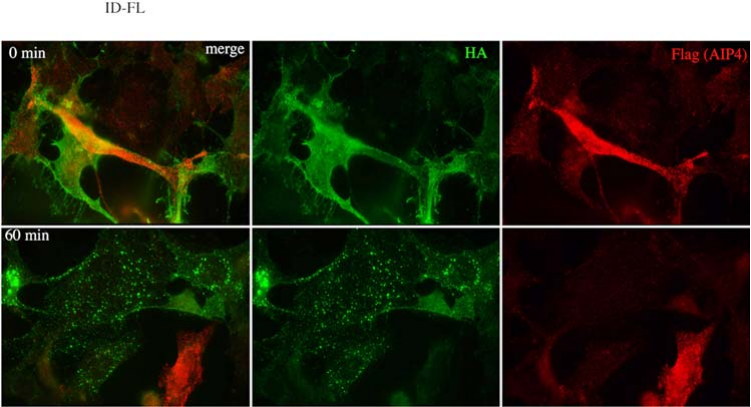
AIP4 complementation in ID-FL cells. The anti-HA antibody uptake assay (see figure 2) was performed on ID-FL cells prealably transfected with Flag-AIP4 expression vector. After 0 or 60 minutes of chase, cells were fixed, permeabilized and incubated with a CY3-coupled anti-Flag antibody.

As the relative quantity of Notch at the membrane was the same in MD-FL and ID-FL cells (Figure S1), we wanted to establish whether Itch regulates the postendocytic sorting of Notch independently of ligand binding. We monitored Notch 1 internalization by an antibody uptake experiment in the absence of any ligand. Notch 1 in MD-FL or ID-FL cells was detected with the use of an extracellular HA epitope (figure 2, 0 min). The cells were incubated with a fluorescently labeled anti-HA antibody at 4°C for 30 minutes, then incubated at 37°C for various periods of time, fixed and observed to track the destination of cell surface-localized Notch. Notch receptor incubation with anti-HA antibody did not provoke Notch activation, since no nuclear staining with anti-activated Notch antibody was observed (data not shown). Furthermore the staining pattern, observed with an anti-Notch receptor after fixation and permeabilization, was the same, whether cells had been treated with anti-HA or not (data not shown). After a 30 min incubation at 37°C, much of the Notch immunoreactivity was detected in intracellular vesicles, suggesting that cell surface Notch is constantly internalized in WT as well as in Itch -/- cells (Figure 2, line 30 min). After 60 and 90 minutes, almost no HA staining was detectable in MD-FL cells (first column), suggesting that Notch or the HA antibody had been degraded. Concomitant treatment with the proteasome inhibitor lactacystin did not affect this kinetics, whereas inhibition of lysosomal proteases by leupeptin treatment prolonged the HA staining at least until 90 minutes of internalization (MD-FL+leupeptin). In contrast, in ID-FL cells, HA staining was visible after 60 or 90 minutes of incubation, irrespective of lactacystin or leupeptin treatment (see quantification, figure 2 bottom). On the other hand, EGFR degradation after EGF activation, monitored in the same type of assay with uptake of fluorescent EGF, was identical after a 60 min incubation time in MD-FL and in ID-FL cells (Figure S2, compare MD-FL in A to ID-FL in B). Thus the ID-FL cells are not impaired in a general lysosomal activity or in the endocytic pathway. These results suggest that HA staining disappearance in MD-FL cells is due to Notch degradation in the lysosomes. In ID-FL cells Notch degradation was strongly delayed. Moreover in these cells the Notch-positive endocytic vesicles, detected at 30 minutes (Figure 2) were more scattered throughout the cells, as compared to MD-FL cells. We confirmed these results by co-labelling LAMP-1 after cell fixation and permeabilisation. At 90 minutes of Notch internalization, the HA-positive vesicles in ID-FL were essentially negative for the lysosomal marker LAMP-1 (Figure 3), whether the cells have been treated with leupeptin or not. When MD-FL cells were treated with leupeptin, Notch accumulated in LAMP-1 positive vesicles, showing that it had reached the lysosomes. These results suggest that Notch targeting and degradation in the lysosomes depends on the activity of Itch. To confirm that the presence of Itch is important for this late targeting, we prepared endocytic fractions from MD-FL and ID-FL cells. Notch full-length molecule (indicated as p300 in figure 4A) was detectable in the post-nuclear supernatant (P in lanes 1, 4, 7, 10), and very poorly in the early endosomal fractions (E, lanes 2, 5, 8, 11), confirming the non-contamination of our preparations with Golgi or endoplasmic reticulum proteins [22]. This was further verified thanks to an anti-GM130 (a Golgi protein) western blotting (Figure 4B). The heterodimeric receptor, detected as p120 with an antibody recognizing the intracellular part of Notch 1, was present in early endosomal fractions (E) in both MD-FL and ID-FL cells, but in late endosomal fractions (L) only in MD-FL cells. These fractions were enriched in p120 when MD-FL cells were treated with leupeptin, confirming Notch 1 lysosomal degradation (4A, lanes 7 to 9). Itch was mainly detected in E fraction, consistent with its role in this compartment (4B, bottom panel). In ID-FL cells, p120 was only detected in E fractions, irrespective of leupeptin treatment (lanes 5–6 and 11–12). On the other hand, EGFR fractionation was similar in both cell lines (Figure 4B), thus validating the preparations. These results are in agreement with Itch being necessary for Notch transition from early to late endosomes, before lysosomal degradation. To further prove this requirement, we transiently complemented ID-FL cells with AIP4 (Figure 5). Notch expression at the membrane was not affected by overexpression of AIP4 (0 min). On the other hand, Notch degradation was visible after 60 minutes of internalization time in AIP4-transfected cells, whereas in the neighbouring non-transfected cells, Notch was still detected in endocytic vesicles. This result shows that AIP4 complementation is sufficient to restore Notch targeting to lysosomal degradation.

### Ubiquitination of Notch is dependent on Itch

**Figure 6:**
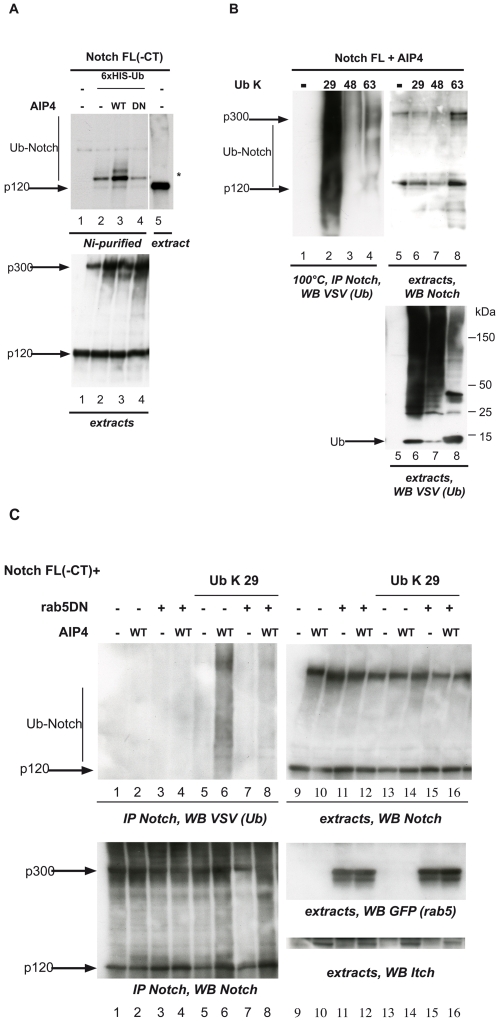
AIP4 stimulates polyubiquitination of Notch through K29-linked chains after early endocytosis. A. HEK293T were transfected with Notch FL-CT, AIP4 WT or DN, and 6×His-ubiquitin expression vectors when indicated. His-Ub-conjugated forms of Notch were purified in denaturing conditions on Ni+-sepharose (lanes 1–4) and tested by immunoblotting with anti-Notch IC. A Notch-containing cell lysate was tested in parallel (lane 5) to visualize the non-ubiquitinated Notch. The * indicates the starting point of the ubiquitinated products derived from Notch. Bottom, the corresponding cell extracts were directly analyzed with the same antibody. B. HEK293T were transfected with Notch FL(HA), AIP4 and VSV-tagged ubiquitin (Ub) expression vectors. The number indicates the only lysine residue remaining in the ubiquitin molecule. Cell extracts were directly tested for Notch (lanes 5–8) or Notch ubiquitinated products were purified via denaturing immunoprecipitation (lanes 1–4) and revealed by anti-VSV western blotting. Bottom, the cell extracts were directly analyzed by SDS-PAGE on a 4–20% gradient gel followed by immunoblotting with anti-VSV antibody, which revealed the free ubiquitin molecules (arrow) and a smear corresponding to the ubiquitinated proteins in the whole extracts. C. HEK293T were transfected with expression vectors encoding for Notch FL(−CT), Ub K29, AIP4 (WT) and a dominant-negative form of rab5 linked to GFP (rab5DN) when indicated. Notch products were purified by immunoprecipitation and the ubiquitinated molecules were revealed by anti-VSV (lanes 1–8) followed by anti-Notch (bottom) western blotting. As controls, cell extracts were directly tested for Notch, rab5 (anti-GFP) and Itch (lanes 9–16).

In order to determine whether Itch acts on Notch through its E3-ubiquitin ligase activity, we monitored Notch ubiquitination by transfecting 293T cells with expression vectors encoding 6×His-tagged Ubiquitin together with Notch FL(−CT) (deleted of the C-terminal domain, amino-acids 1 to 2183 of Notch1, see [23]), AIP4 or an inactive form of AIP4, carrying a mutation in the active site (named AIP4DN). Ni2+-chelating sepharose was used to purify the ubiquitinated conjugates in denaturing conditions (8M urea), and the ubiquitination levels of Notch were analyzed by immunoblotting. When 6×HIS-Ub was coexpressed (Figure 6A, lanes 2–4), we specifically detected a monoubiquitinated Notch species (indicated by *), as well as a smear representing the polyubiquitinated forms (indicated by Ub-Notch). Overexpressing AIP4 enhanced Notch polyubiquitination (lane 3), whereas AIP4DN overexpression severely impaired it (lane 4). To identify the type of isopeptide linkage catalysed on Notch, we made use of expression vectors encoding VSV-tagged ubiquitins, allowing the formation of a single type of polyubiquitin chains [12]. After 293T cells transfection with Notch FL(HA) (which contains the carboxy-terminal sequence of Notch1), AIP4 and ubiquitin expression vectors, extracts were boiled in SDS-containing buffer, before immunoprecipitation with anti-Notch antibody and detection of the ubiquitinated products with anti-VSV. Ubiquitination of Notch was predominantly detected when using Ub K29, as compared with Ub K48 or K63 (Figure 6B, lanes 2–4). The fact that polyubiquitinated products were detected in these denaturing conditions strongly suggests that they are Notch products. We verified by direct western blotting of the extracts that all ubiquitin vectors allowed the expression and incorporation of the mutant ubiquitins (Figure 6B, bottom). Finally, when monitoring EGFR ubiquitination after EGF treatment, all ubiquitin vectors, including Ub K0 (without any lysine) allowed the same smear to be detected, showing that multiubiquitination was mostly produced, as expected ([24], Figure S3). This confirmed that preferential K29 ubiquitination was not an artifact of our VSV-Ub vectors. The fact that Notch polyubiquitination was detected with these VSV-ubiquitin constructs, whereas monoubiquitination was predominant when using His-Ub was an artifact of the His-Ub constructs, that we observed with various ubiquitination substrates and that may be due to steric hindrance caused by the 6×His tag. Furthermore the effect of AIP4 WT or DN on Notch (− or + CT) ubiquitination was also seen with the VSV-ubiquitin expression vectors (Figure S4). However only a mass spectrometry analysis of Notch products would allow the identification of the ubiquitination sites on Notch and of the major type of chains formed. Taken together, our results show that Notch, in the absence of ligand, represents a new substrate of AIP4, submitted to the same modification that we have described for Deltex [12].

In order to precisely identify the step where ubiquitination of Notch by Itch takes place, we monitored Notch FL(−CT) ubiquitination when early endocytosis was inhibited by overexpressing a dominant negative form of rab5 (rab5DN, fused to GFP). As shown in figure 6C, Itch-dependent, K 29-linked polyubiquitination (lanes 5–6) was largely diminished in the presence of rab5DN (lanes 7–8), although Notch quantities were comparable in the extracts (lanes 9–16) and in the immunoprecipitates (lanes 1–8, bottom). We concluded that Itch ubiquitinates Notch after early endocytosis.

### Interaction between Notch and Itch is not direct

**Figure 7:**
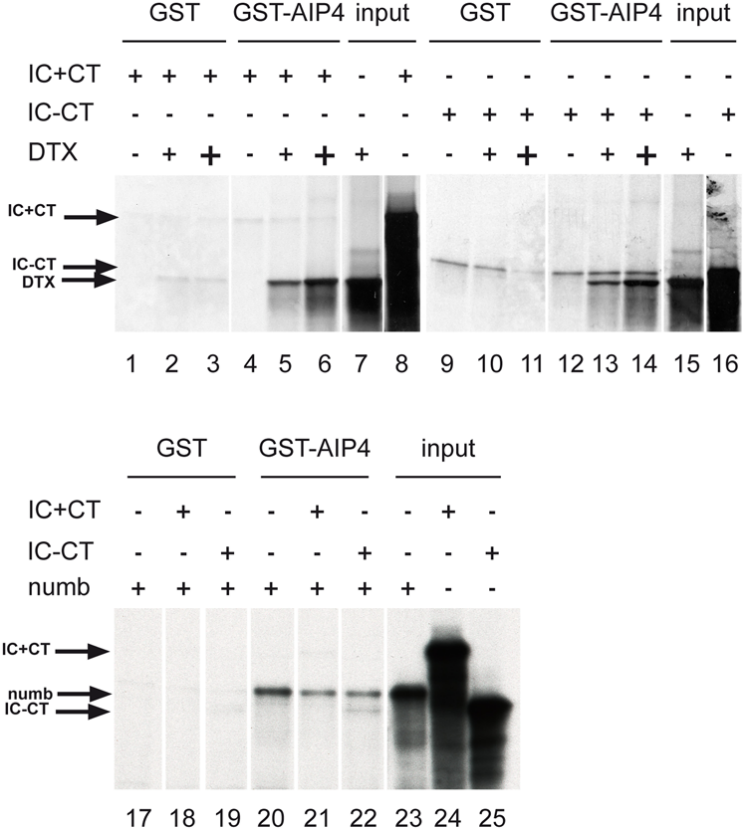
Notch does not interact directly with Itch/AIP4. The Notch IC+CT, IC-CT, numb and DTX proteins were in vitro-translated in the presence of ^35^S met, and their ability to be retained onto a GST-AIP4 fusion protein or control GST adsorbed to glutathione-agarose beads was analyzed by SDS-PAGE analysis. The input lanes (7, 8, 15, 16, 23, 24, 25) show the different in vitro-translated products prior to incubation with the beads. Two concentrations of in vitro translated DTX (0.5 or 3 µl, indicated by + and + respectively) were tested. White lines indicate that intervening lanes have been spliced out.

In Drosophila Su(dx) and Notch interact directly through their WW and PPSY motif respectively [18], [19], [25]. The PPSY interaction motif of Drosophila Notch is transformed into PPRL in mammals (aa 2260–2263 in Notch 1), a sequence which is absent in the (−CT) form of Notch. However the presence of the C-terminal domain of hNotch 1 is not necessary for Itch to ubiquitinate Notch (see figure 6A and C). On the other hand Qiu et al [20] have concluded that Itch binds to the N-terminal portion of Notch intracellular domain. We thus examined whether the interaction between Itch and Notch was direct, using GST fusion proteins containing fragments of AIP4 bound to gluthatione-agarose beads, and incubated with translation products obtained in rabbit reticulocyte lysates. We used IC forms of Notch (− and + CT) to avoid the conformation problems due to the presence of transmembrane domains. Figure 7 shows that the IC forms of Notch could not efficiently interact with AIP4 in vitro (lanes 1, 4, 9, 12). Thus a post-translational modification of Notch or Itch might be necessary to allow recognition. Alternatively it is possible that the interaction needs a cofactor. We tested several proteins as possible bridging factors between Notch and AIP4 in this test assay. DTX (two concentrations, indicated with small or large + in lanes 2–7 and 10–15) and numb (lanes 17–22) (see Discussion) were specifically retained on GST-AIP4 in the presence or absence of ICs. In spite of their presence, Notch was not better pulled-down by GST-AIP4 (lanes 5, 6, 13, 14, 21, 22). The same results were obtained when testing β-arrestin 1 or 2 (data not shown).

**Figure 8:**
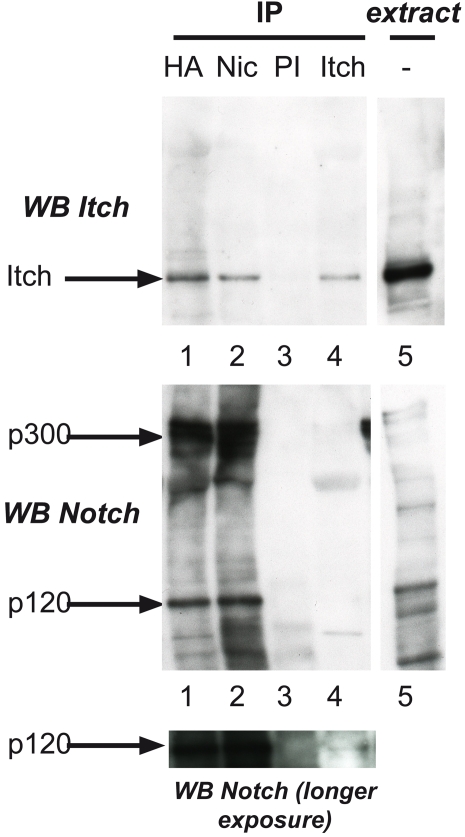
AIP4/Itch associates with Notch in non-activated cells. Digitonin solubilized extracts from MD-FL cells were immunoprecipitated with antibodies for Notch EC (anti-HA antibody, lane 1), Notch IC (lane 2), a rabbit preimmune serum (PI, lane 3) or rabbit anti Itch (lane 4). The immunoprecipitates were analyzed by SDS-PAGE followed by immunoblotting with monoclonal anti-Itch and anti-Notch as indicated. Lane 5 contains an aliquot of the starting material. Bottom panel shows a longer exposure of the anti-Notch blot allowing detection of p120 in lane 4.

Notch and Itch overexpression and coimmunoprecipitation is not a conclusive assay to demonstrate that Notch interacts with Itch during its trafficking. Hence we decided to purify Notch and Itch-containing complexes from MD-FL cells. We used a large panel of detergents to solubilize membrane proteins, and notably only digitonin allowed specific co-immunoprecipitation of Itch with Notch (Figure 8). This was true when using antibodies directed against either the extracellular part of the receptor (anti-HA, lane 1) or its intracellular domain (Nic, lane 2), suggesting an interaction with the heterodimeric form of Notch. In addition a long exposure of anti-Itch immunoprecipitate immunoblotted with anti-Notch allowed the detection of the p120 form (lane 4 of bottom panel). Taken together, our results suggest that Itch interacts indirectly with heterodimeric Notch in the cells.

## Discussion

### Role of Itch/AIP4 in Notch signaling

Modulating the amount of Notch receptor that is available for signaling could provide one of the mechanisms to finely tune the activity of the pathway. Therefore ubiquitination events and endocytic trafficking of the receptor could constitute key events, particularly in the light of the results showing that mutations that compromise sorting of ubiquitinated membrane protein (ESCRT components for instance) result in overactivation of Notch [26]–[28]. Our results show that, in the absence of any activation, mammalian Notch receptor is constitutively internalized and degraded in the lysosomes. Notch degradation in the lysosomes is abolished by treating the cells with leupeptin, whereas a proteasomal inhibitor, lactacystin does not affect this event. Thus the regulation of the quantity of functional Notch receptor occurs mostly through its lysosomal degradation. This result seems contradictory to what was proposed by others, who observed an accumulation of drosophila Notch receptor in the presence of MG101 or MG132 [18], [29]. However these proteasome inhibitors, unlike lactacystin, could also inhibit lysosomal Cys proteases [30], or they could act indirectly on Notch stability. Recycling of Notch receptor is not completely ruled out, in particular in the Itch -/- cells, but this type of event should have been detected in our antibody uptake experiment where the antibody used did not dissociate from the receptor in a moderately acidic environment.

We show here that the initial steps of Notch endocytosis do not depend on Itch/AIP4. However this E3 ubiquitin ligase is required at a later step allowing final targeting to the lysosomes, since we observe that Notch is still internalized in Itch-/- cells and accumulates in endosomes that are adjacent to LAMP-1 positive vesicles and that ubiquitination is impaired when early endocytosis is blocked. It should be mentioned that although Notch degradation is severely delayed in Itch -/- cells, other members of the Itch family such as Nedd4 are still present in these cells and may partially replace it. Nevertheless the reintroduction of AIP4 in ID-FL cells is required to fully restore the kinetics of Notch degradation. We also propose that AIP4/Itch mainly catalyses the formation of polyubiquitin chains through the lysine 29 of ubiquitin, as is the case for two of its substrates, DTX and itself [12]. Hence Notch seems to behave like DTX towards AIP4, both regarding the type of isopeptide linkage used and the localisation and time of the ubiquitination events in the endocytic pathway.

It should be mentioned that other ubiquitination events affecting Notch and catalyzed by other E3 ubiquitin ligases could happen before or after Itch requirement. The RING E3 ubiquitin ligase c-cbl, which was described to be necessary for Notch lysosomal degradation [31] could fulfill this function. On the other hand, other receptors have been recently described to be endocytosed without any ubiquitination event, this step taking place later. This was recently demonstrated for EGFR, where mutating all lysine residues in the kinase domain did not affect the internalization rate of the receptors [32]. More complicated situations may be envisaged, as recently shown for the interferon receptor IFNAR1, where ubiquitinations through K48 and K63 linkages on specific sites are required for the receptor to be internalized after ligand stimulation, the efficiency of this process being affected by the ubiquitination-dependent exposure of a linear endocytic motif present in the cytoplasmic tail of the receptor [33]. Therefore mechanisms underlying Notch receptor endocytosis are not completely understood yet, although its ubiquitination through K29 chains represents a signature of a very specific Itch-dependent spatiotemporal step.

### Parallels and Differences between drosophila Su(dx) and mammalian Itch/AIP4

The Drosophila Su(dx) gene was described as a negative regulator of Notch signaling [2]–[4]. It has been shown that Su(dx) and Nedd4 regulate the postendocytic sorting of Notch en route to the late endosome [18], [19]. Our data confirm and extend these observations by identifying the way Notch is degraded and the step regulated by Itch. Nedd4 suppression in drosophila leads to Notch-dependent and ligand-independent activation of Notch target genes [18], possibly by stabilizing the Notch-Dx complex and facilitating γ-secretase proteolytic cleavage of Notch. We did not observe any constitutive activation of Notch in Itch -/- cells, although Notch was fully activable by EDTA treatment even in the absence of Itch. This discrepancy might be related to the different protein complexes formed in drosophila and in mammals. Indeed it has been well documented that Su(dx) WW domains directly interact with a PPSY motif in the drosophila Notch intracellular domain [18], [19], [25]. Our data using GST pull-down experiments show that unmodified forms of Notch or AIP4 cannot stably interact together. Moreover the crucial tyrosine present in the interaction motif of Drosophila Notch is absent in mammalian Notch, and the presence of the C-terminal part of mammalian Notch does not affect its ability to be ubiquitinated by Itch. Taken together, these results suggest that mammalian Notch does not use such a motif to interact with Itch. Therefore the protein complexes containing Notch and Su(dx) in drosophila might be different from those containing Notch and Itch/AIP4 and found during endocytosis in mammals.

Qiu et al [20] concluded that murine Itch binds to the N-terminal portion of Notch intracellular domain, although they did not prove that the interaction was direct. We were able to co-immunoprecipitate endogenous Notch and Itch only when solubilizing membrane complexes with the mild detergent digitonine. Therefore such membrane Notch-containing complexes might also contain other proteins, the presence of which is necessary to connect Notch and Itch. The same type of events have been described in mammals for other HECT-E3 ubiquitin ligases. In particular after TGFβ stimulation, Smurf1 and Smurf2 participate in the ubiquitination and degradation of TGFβ receptor and SnoN, through their association with Smad7 and Smad2 respectively [34]–[36].

Based on genetical and biochemical data, we tested in vitro two possible bridging factors between Itch and Notch, DTX and numb. DTX in mammals has been shown to interact with Itch [12] and Notch [37], and is associated with endocytic vesicles [12]. Numb was proposed to promote Notch receptor ubiquitination and degradation of its intracellular domain after activation [38]. On the other hand, numb targets Gli1 for Itch-dependent ubiquitination [39]. Although both proteins could constitute good candidates, their presence did not improve Notch binding to GST-AIP4. Finally we tested beta-arrestin 1 and 2, since β-arrestin 1 was very recently demonstrated to be necessary to promote the interaction between AIP4 and activated CXCR4 [40]. Furthermore β-arrestin 2 drosophila ortholog, Krz, was described as a negative regulator of Notch signaling acting in a deltex-dependent manner [29]. However these factors did not facilitate Notch binding to AIP4 in vitro. Therefore we conclude that either other factors or post-translational modifications are necessary for the Notch-Itch interaction.

### What happens when Notch is activated?

Our results show that activation of Notch FL is not affected by the presence of Itch. However both types of fates, Itch-dependent Notch degradation on one hand, ligand-dependent Notch activation on the other hand, rely on endocytosis of the receptor [41]. One can imagine two possibilities: in the first model, Itch is associated with Notch complexes at the membrane, then ligand activation and ADAM cleavage produce a new form which can no longer interact with Itch, thus allowing Notch sorting to different vesicles where the interaction with γ-secretase elements and subsequent cleavage can occur. Alternatively Itch association occurs after sorting of activated Notch to the vesicles where it is cleaved by the γ-secretase complex, and thus can happen only by default on the receptor molecules which have not been activated. This hypothesis is however difficult to reconcile with the fact that Itch colocalizes with Hrs-positive vesicles [7], [12], [40] and that γ-secretase cleavage seems to occur downstream of these vesicles [41]–[43]. Irrespective of the model, further experiments are required to characterize the discriminating events and the partners involved in the sorting.

## Materials and Methods

### Reagents, constructs and cellular biochemical studies

Antibodies were used at the indicated dilutions for western blot and supplied by Santa Cruz (anti-EGFR, dilution 4000), BD Transduction Laboratories (monoclonal anti-Itch and anti-GM130, diluted respectively 3000 and 800 fold), Abgent (polyclonal anti-Itch), Sigma (anti-Flag M2), Molecular Probes, Inc. (Alexa Fluor® conjugates), Invitrogen (rabbit anti-GFP, dilution 5000), Cell Signaling (anti Notch V1744, dilution 2000) or provided by J.T. August (anti-LAMP1, obtained from the Developmental Studies Hybridoma Bank developed under the auspices of the NICHD and maintained by the University of Iowa, Department of Biological Sciences, Iowa City, IA 52242). Anti-Notch IC antibody was described in [22]. Expression vectors were gifts of A. Atfi (INSERM U482, Paris, France, AIP4 and AIP4DN (C830A)), J. Aster (Harvard Medical School, Boston, USA, Notch FL(HA) retroviral vector), S. Conner (The Scripps Research Institute, La Jolla, USA, numb), R. Kopan (Washington University, St Louis, USA, Notch IC), M. Treier (EMBL, Heidelberg, Germany, 6×HIS-Ub), M. Zerial (Max-Planck-Institute of Molecular Cell Biology and Genetics, Dresden, Germany, rab5DN-GFP). GST-AIP4 was obtained from A. Angers (Université de Montréal, Canada). DAPT was from Calbiochem. Lactacystin and Leupeptin were from Sigma and used respectively at 10 and 100 µM.

MD-FL and ID-FL cell lines were established by retroviral transduction: High titers of recombinant Notch FL(HA) viruses were obtained 48 h after transfection of the Plat-E ecotropic packaging cell line with retroviral expression plasmids (gifts of J. Aster, Harvard medical school, Boston, USA). After retroviral transduction of the MD and ID cell lines (Mouse embryonic fibroblasts derived from WT or Itch-/- animals [12]), clonal populations were obtained by limiting dilution.

Transfections: Ca-phosphate for HEK293T, Fugene 6 (Roche) for Plat-E, Fugene HD (Roche) for MEF cell lines, according to manufacturer's instructions.

Cell cultures, immunofluorescence, immunoprecipitations and immunoblots experiments were performed as previously described [12]. For immunofluorescence, images were acquired with 0,3 µm sections using an Axioplan 2 imaging with Apotome system (Carl Zeiss MicroImaging Inc., Le Pecq, France). The magnification for all photographs was 63×.

### Ubiquitin-conjugates purification

Ni sepharose purification: 293T cells were harvested 24 h after transfection and lysed in 8 M urea, 0.1 M NaH2PO4, 10 mM Tris-Hcl (pH 8), 1% Triton X-100 and 20 mM Imidazole at room temperature. His-Ub conjugated proteins were purified on chelating Sepharose beads (Pharmacia), prealably charged with Nickel. Ni-bound proteins were washed extensively with the same buffer, then with a pH 6.3 buffer and eluted in Laemmli before western blot analysis.

Denaturing immunoprecipitation: 293T cells were collected 24 h after transfection, washed in PBS buffer and lysed in 50 mM Tris-Hcl (pH 8.0), 1% Triton X-100, 150 mM NaCl supplemented with 1×protease inhibitor cocktail (Roche) and 5 mM N-ethylmaleimide (Sigma). After removing of the insoluble material by centrifugation, extracts were boiled in 1% SDS for 5 minutes. After neutralization of SDS by 10-fold dilution in Triton buffer, immunoprecipitation was performed.

### HA antibody or EGF uptake assays

After washing the cells in serum-free medium, they were incubated at 4°C for 30 min with anti-HA-Al488 (or a mixture with EGF-Al555), then washed again and incubated in serum-free medium for various periods of time at 37°C. The cells were then quickly rinsed in cold PBS, fixed and processed for immunostaining. If stained additionally with anti-LAMP-1, this was applied after the permeabilization step.

### Suborganellar fractionation

The preparation of early and late endosomal enriched fractions from MD-FL or ID-FL cells was performed after 16 h of leupeptin treatment according to [44]. Briefly, cells were washed and harvested in ice-cold PBS, pelleted and resuspended in 250 mM Sucrose, 20 mM Tris pH 7.9, 3 mM Imidazole. Cells were broken with a dounce (pestle B), the post nuclear supernatant (P) was collected after centrifugation at 3000 rpm for 10 min. It was then brought to 40.6% sucrose, loaded at the bottom of an ultracentrifuge tube, and sequentially overlaid with 35%, 25% and 8.6% sucrose containing buffers. The gradient was centrifuged at 35000 rpm for one hour at 4°C in a SW55Ti rotor. Early and late endosomal fractions were collected at the 35/25 and 25/8.6% sucrose interfaces respectively.

### Activation by EDTA treatment

Notch heterodimer dissociation was obtained as described [21]. After washing with HBSS (Gibco-BRL), the cells were incubated in pre-warmed 10 mM EDTA-containing HBSS for 15 min at 37°C. The medium was replaced by PBS and cells were directly collected and lysed. When necessary, DAPT (5 µM) was added 1 h before activation and maintained during the treatments.

### GST pull-down analysis

In vitro-translated proteins were synthesized in a reticulocyte lysate-coupled transcription/translation system (Promega), in the presence of ^35^S methionine. Approximately equal amounts of glutathione-S transferase (GST) alone or in fusion with AIP4 [17], as estimated from a Coomassie-stained gel, were bound to glutathione-Agarose (Sigma). The in vitro translations were incubated for 2 hours at 4°C with these beads in 1% Triton-containing buffer. The beads were then extensively washed in the same buffer and the bound proteins were eluted by boiling in Laemmli buffer, subjected to SDS-PAGE analysis followed by fluorography.

## Supporting Information

Figure S1 MD-FL and ID-FL cells exhibit similar amounts of Notch at the membrane: Surface proteins of both cell lines were labelled with NHS-biotin for 1 hour at 4°C (lanes +). Whole cell extracts (lanes 1–4) and fractions retained on streptavidin-agarose (lanes 5–8) were analyzed by immunoblotting with anti-Notch. Note that only p120 is detected in lanes 6 and 8, confirming the non-contamination with intracellular Notch. As a positive control, EGFR was detected at the membrane, and GM130 was used as a negative control. Immunoreactivity of the upper blot was quantified using Quantity One software (Biorad Lab.). For each cell line, the relative amount of membrane Notch was calculated as the ratio between «streptavidin-purified» and «extract» signals. It was estimated to 3.5% in MD-FL cells and 3.3% in ID-FL cells.
(1.10 MB TIF)

Figure S2 Activation of EGFR in MD-FL and ID-FL cells. Anti-HA antibody (green) and EGF (red) uptake assays were performed concomitantly in MD-FL (A) or ID-FL (B) cells. Insets represent enlargements (eightfold) of the boxed regions.
(5.62 MB TIF)

Figure S3 Ubiquitination of EGFR: HEK293T were transfected with various Ub vectors (as in figure 6B). Ub K0 indicates a mutant where all lysine residues have been replaced by arginine. 24 hours after transfection, cells were treated with EGF for 5 minutes, extracts were prepared and immunoprecipitated with a mouse anti-EGFR (Santa Cruz sc-120). Ubiquitinated products were revealed by western blotting with anti-VSV antibody and controlled by western blotting with rabbit anti-EGFR (sc-03). A similar smear was observed with all ubiquitin constructs, even K0, in accordance with published data showing that multiubiquitination was mostly produced after EGFR activation.
(0.94 MB TIF)

Figure S4 K29-linked polybiquitination of Notch is increased by cotransfected AIP4 and inhibited by AIP4DN. HEK 293T were transfected with expression vectors encoding Notch FL(HA), AIP4, AIP4DN and Ub K29 as indicated. The experiment was performed as in figure 6B. Immunoprecipitates and extracts were respectively analyzed by western blotting with anti-VSV and anti-Notch.
(0.44 MB TIF)
