# Supplementary figures and images for: AIP4/Itch Regulates Notch Receptor Degradation in the Absence of Ligand
Source: PLoS One. 2008 Jul 16;3(7):e2735. doi: 10.1371/journal.pone.0002735 (PMC2444042; doi:10.1371/journal.pone.0002735)

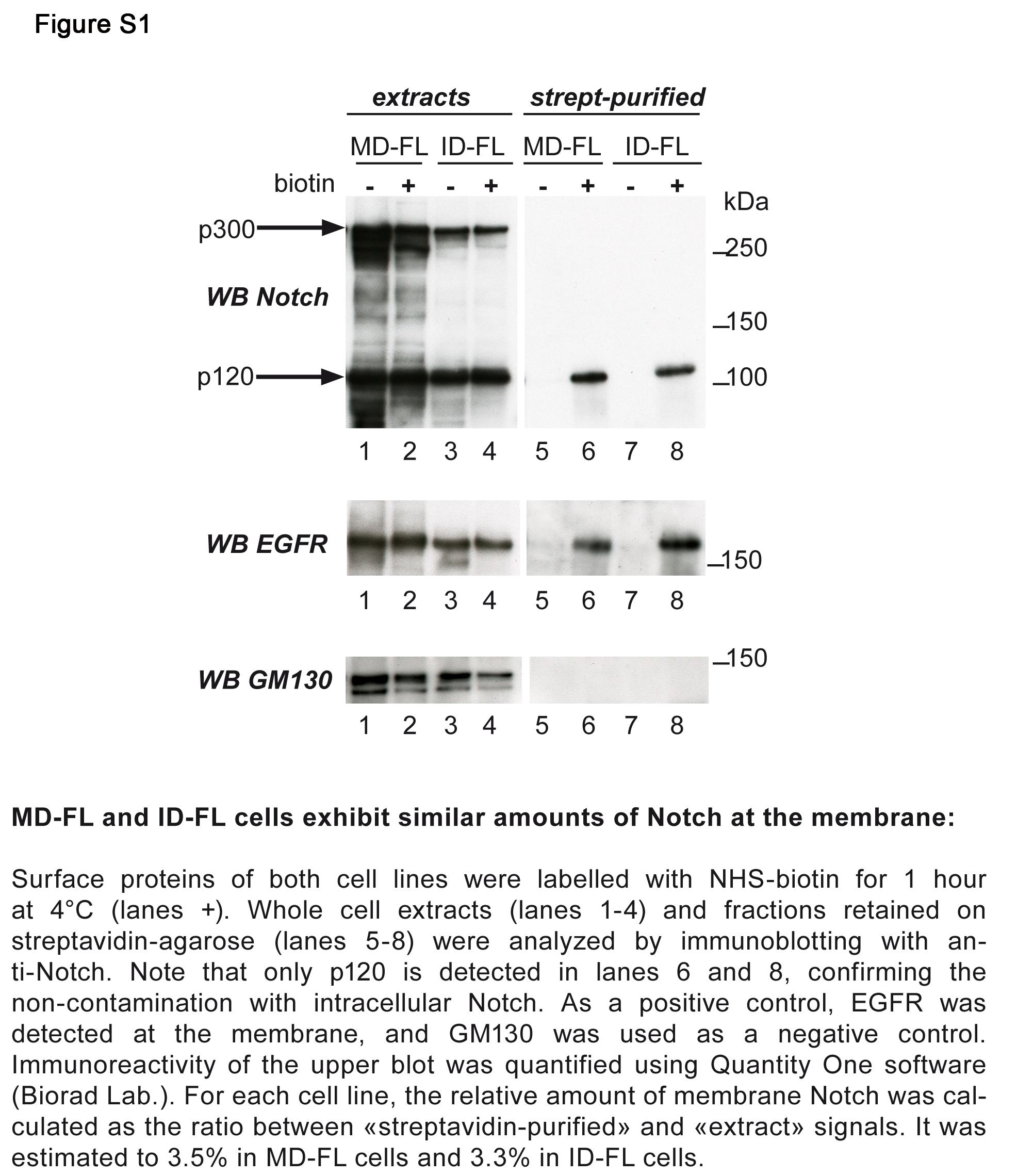

Supplement: Figure S1 — MD-FL and ID-FL cells exhibit similar amounts of Notch at the membrane: Surface proteins of both cell lines were labelled with NHS-biotin for 1 hour at 4°C (lanes +). Whole cell extracts (lanes 1–4) and fractions retained on streptavidin-agarose (lanes 5–8) were analyzed by immunoblotting with anti-Notch. Note that only p120 is detected in lanes 6 and 8, confirming the non-contamination with intracellular Notch. As a positive control, EGFR was detected at the membrane, and GM130 was used as a negative control. Immunoreactivity of the upper blot was quantified using Quantity One software (Biorad Lab.). For each cell line, the relative amount of membrane Notch was calculated as the ratio between «streptavidin-purified» and «extract» signals. It was estimated to 3.5% in MD-FL cells and 3.3% in ID-FL cells. (1.10 MB TIF) [file pone.0002735.s001.tif]

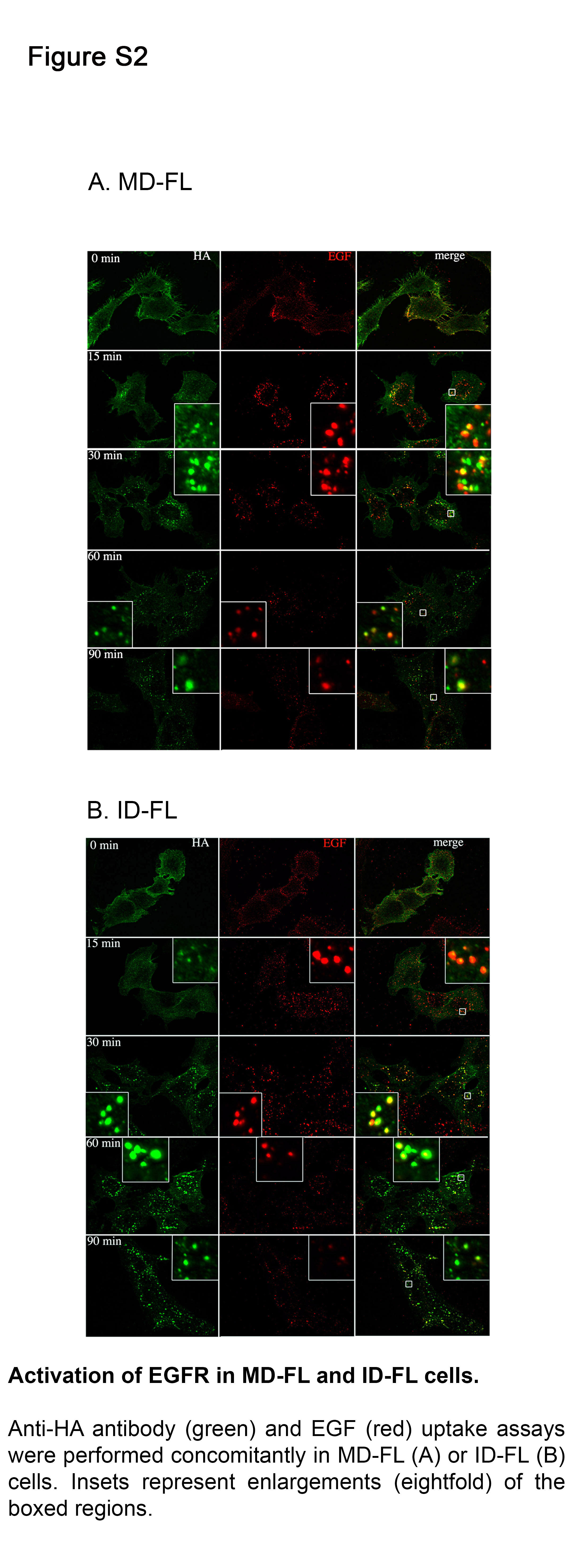

Supplement: Figure S2 — Activation of EGFR in MD-FL and ID-FL cells. Anti-HA antibody (green) and EGF (red) uptake assays were performed concomitantly in MD-FL (A) or ID-FL (B) cells. Insets represent enlargements (eightfold) of the boxed regions. (5.62 MB TIF) [file pone.0002735.s002.tif]

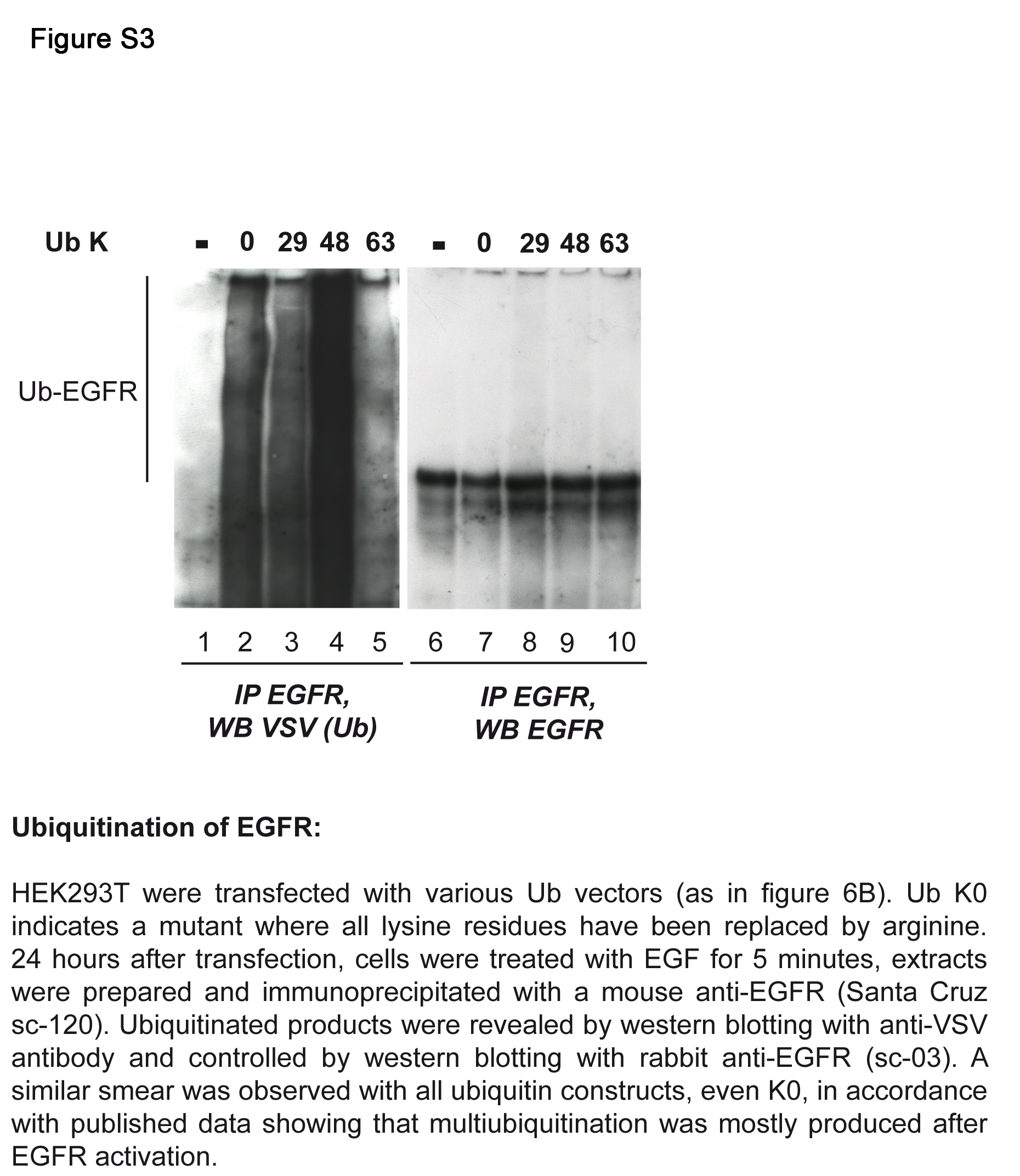

Supplement: Figure S3 — Ubiquitination of EGFR: HEK293T were transfected with various Ub vectors (as in figure 6B). Ub K0 indicates a mutant where all lysine residues have been replaced by arginine. 24 hours after transfection, cells were treated with EGF for 5 minutes, extracts were prepared and immunoprecipitated with a mouse anti-EGFR (Santa Cruz sc-120). Ubiquitinated products were revealed by western blotting with anti-VSV antibody and controlled by western blotting with rabbit anti-EGFR (sc-03). A similar smear was observed with all ubiquitin constructs, even K0, in accordance with published data showing that multiubiquitination was mostly produced after EGFR activation. (0.94 MB TIF) [file pone.0002735.s003.tif]

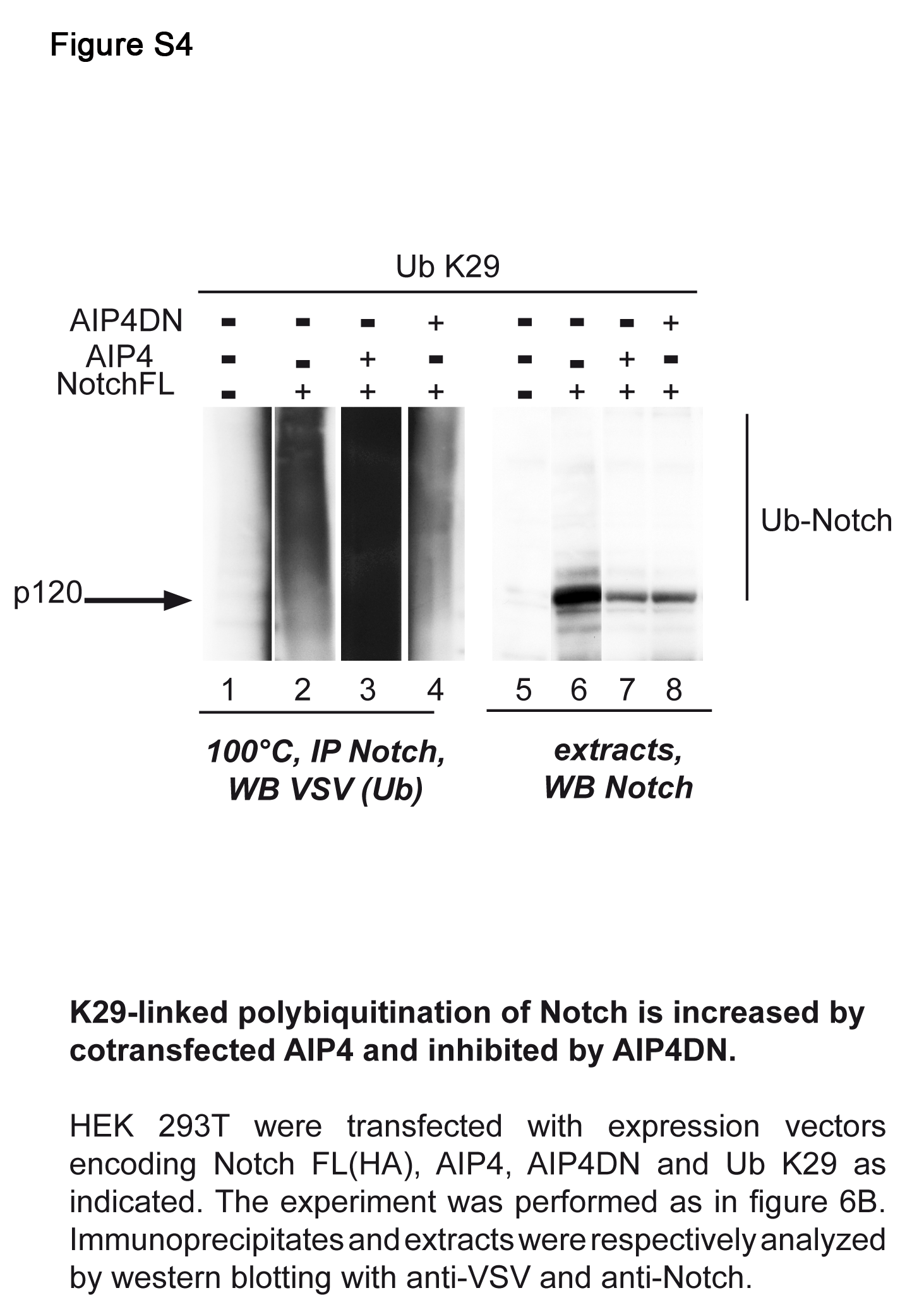

Supplement: Figure S4 — K29-linked polybiquitination of Notch is increased by cotransfected AIP4 and inhibited by AIP4DN. HEK 293T were transfected with expression vectors encoding Notch FL(HA), AIP4, AIP4DN and Ub K29 as indicated. The experiment was performed as in figure 6B. Immunoprecipitates and extracts were respectively analyzed by western blotting with anti-VSV and anti-Notch. (0.44 MB TIF) [file pone.0002735.s004.tif]
